# Systematic or On-Demand Sealant Use in Minimally Invasive Lung Surgery: A Matched Comparison

**DOI:** 10.1093/icvts/ivag094

**Published:** 2026-03-28

**Authors:** Stefano Rudella, Clara Brezillon, Elsa Armand, Alban Todesco, Vanessa Hubaud, Geoffrey Brioude, David Boulate, Delphine Trousse, Zeinab Hamidou, Mohamed Boucekine, Christophe Doddoli, Florence Peyron, Pascal-Alexandre Thomas, Alex Fourdrain, Xavier Benoit D’Journo

**Affiliations:** Department of Thoracic Surgery, North Hospital, Aix-Marseille University, Marseille, 13915, France; Departement of Pharmacy, North Hospital, Aix-Marseille University, Marseille, 13915, France; Department of Thoracic Surgery, North Hospital, Aix-Marseille University, Marseille, 13915, France; Department of Thoracic Surgery, North Hospital, Aix-Marseille University, Marseille, 13915, France; Department of Thoracic Surgery, North Hospital, Aix-Marseille University, Marseille, 13915, France; Department of Thoracic Surgery, North Hospital, Aix-Marseille University, Marseille, 13915, France; Department of Thoracic Surgery, North Hospital, Aix-Marseille University, Marseille, 13915, France; Department of Thoracic Surgery, North Hospital, Aix-Marseille University, Marseille, 13915, France; Departement of Biostatistics, CEReSS, Aix-Marseille University, Marseille, 13915, France; Departement of Biostatistics, CEReSS, Aix-Marseille University, Marseille, 13915, France; Department of Thoracic Surgery, North Hospital, Aix-Marseille University, Marseille, 13915, France; Departement of Pharmacy, North Hospital, Aix-Marseille University, Marseille, 13915, France; Department of Thoracic Surgery, North Hospital, Aix-Marseille University, Marseille, 13915, France; Department of Thoracic Surgery, North Hospital, Aix-Marseille University, Marseille, 13915, France; Department of Thoracic Surgery, North Hospital, Aix-Marseille University, Marseille, 13915, France

**Keywords:** lung surgery, air leak, sealant

## Abstract

**Objectives:**

Prolonged air leak remains a major concern following minimally invasive lung cancer surgery. This study aimed to evaluate whether the systematic use of aerostatic sealants improves postoperative outcomes compared to an on-demand strategy.

**Methods:**

Over a 12-month period, we selected patients underwent anatomical lung resection with minimally invasive techniques for lung cancer. Before March 2023, patients received an absorbable polyglycolic acid patch selectively based on intraoperative findings (on-demand group). Thereafter, a cohort of consecutive patients received a polyethylene glycol sealant systematically (systematic group). Patients matched 1:1 using a propensity score based on type of resection, surgical approach, Forced Expiratory Volume, Chronic Obstructive Pulmonary Disease and Diffusing Capacity of the Lung. The primary end-point was length of hospital stay. Secondary end-points included duration of chest tube drainage, redrainage or blood patch, postoperative complications, and 90-day readmission.

**Results:**

Two hundred and thirty patients were selected, 164 in on-demand group, and 66 in systematic group; After matching, there were 56 patients in each group. Preoperative baseline characteristics were similar between group. Sealants were used in 100% of patients in the systematic group versus 13% in the on-demand group (*N* = 29). Median hospital stay was not significantly different (5 [IQR 4-8] vs 5.5 [IQR 3-8] days, *P* = 0.371). No differences were found in mean drainage duration (4 vs 4 days, *P* = .327), complication rate (36% vs 34%, *P* = .491), redrainage (8.9% vs 7.1%; *P* = .999), or prolonged drainage (13% vs 14%, *P* = .781). Ninety-day readmission was not significantly different between groups (11% vs 2%, *P* = .113).

**Conclusions:**

This study revealed no statistically significant differences between the 2 strategies.

## Graphical abstract

**Figure:**
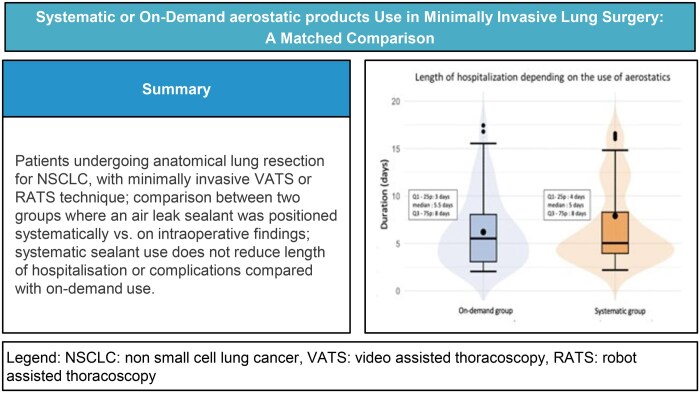

## INTRODUCTION

Postoperative air leak remains one of the most common complications following anatomical lung resection for non-small cell lung cancer (NSCLC), with reported incidence rates ranging from 5% to 25% in the immediate postoperative period.^1^ It is defined as the escape of air from the lung parenchyma, typically through suture lines or areas of fragile tissue that were inadequately sealed during surgery.^2^ While early, transient air leaks are often considered a physiological response to lung re-expansion, those persisting beyond 5-7 days are associated with significantly worse outcomes. These include pleural infections, delayed recovery of pulmonary function, prolonged chest tube drainage, extended hospital stays, and increased healthcare costs—all of which can negatively impact patient quality of life.^3^^,^^4^ Management strategies for postoperative air leak primarily rely on conservative measures, including continued chest tube drainage and, in selected cases, autologous blood patching.^4^ However, persistent or complex air leaks may necessitate more invasive interventions, such as surgical re-exploration, pleurodesis by talc poudrage, or bronchoscopic closure techniques.^5^^,^^6^

To mitigate the incidence and duration of air leaks, a variety of pleural sealants have been developed and are increasingly applied intraoperatively. These include fibrin-based, collagen-based, and polyethylene glycol-based compounds, which are designed to reinforce bronchial stumps or seal areas of parenchymal fragility.^7^^,^^8^ Several randomized trials and systematic reviews have demonstrated their potential to reduce air leak duration and chest tube indwelling time in selected populations.^9^^,^^10^ Nonetheless, the widespread routine and systematic use of such sealants remain controversial, as evidence regarding their cost-effectiveness and impact on broader clinical outcomes remains inconclusive compared to a selective, on-demand application based on intraoperative assessment.^11^^,^^12^

In this context, this study aims to compare clinical outcomes associated with systematic versus selective use of aerostatic sealants during minimally invasive anatomical lung resections for NSCLC, with the goal of guiding clinical decision-making and optimizing postoperative management.

## MATERIAL AND METHODS

### Patients and study design

This study was approved by the ethic committee of the French Society of Thoracic and Cardiovascular Surgery (IRB00012919). Written informed consent for study participation was obtained during the preoperative outpatient consultation, during which the surgeon provided patients with comprehensive information regarding the planned procedure.

We conducted a single-center analysis comparing 2 cohorts of consecutive patients who underwent anatomical lung resections—either lobectomies or segmentectomies—for suspected or documented NSCLC, using minimally invasive techniques: video-assisted thoracoscopic surgery (VATS) or robot-assisted thoracic surgery (RATS). The first cohort, referred to as the On-Demand Group, included consecutive patients operated on between June 2022 and February 2023, in whom intraoperative use of aerostatic sealants was applied selectively, at the discretion of the operating surgeon. This cohort was retrospectively analysed. The second cohort, referred to as the Systematic Group, comprised consecutive patients who underwent surgery between March 2023 and June 2023, in whom aerostatic sealants were applied routinely, regardless of intraoperative findings. This cohort was prospectively analysed and informed consent had been obtained for all patients. All patients were preoperatively evaluated in a multidisciplinary tumour board, which recommended anatomical resection with systematic lymph node dissection as the most appropriate curative treatment for confirmed or suspected early-stage NSCLC. Patients were excluded if they had a neuroendocrine histology (e.g., carcinoid tumors, small cell lung cancer) or a benign lesion, if they underwent open thoracotomy or conversion to thoracotomy, or received a wedge resection.

### Aerostatic used in the study

In the On-Demand Group, intraoperative application of one dose of an absorbable polyglycolic acid patch (Neoveil or equivalent) was performed at the discretion of the operating surgeon. The decision to apply the sealant was based on intraoperative factors, such as a positive hydro-pneumatic test, fragile lung parenchyma, or the presence of significant pleural adhesions. In contrast, the Systematic Group received routine application of one dose of a polyethylene glycol-based surgical sealant (Progel or equivalent) directly to the resection margin, irrespective of intraoperative findings.

The hydro-pneumatic test was performed after completion of the resection. The pleural cavity is partially filled with sterile saline solution, and the lung is gradually inflated under controlled airway pressure. The presence of air bubbles emerging from the staple line or the lung surface indicates an alveolar air leak.

Polyglycolic acid felt and polyethylene glycol-based surgical sealant were applied according to their specific indications and standardized institutional protocols. The first one was used to reinforce the staple line or suture line following lung resection, particularly in cases with fragile parenchyma or anticipated postoperative air leakage. The material was tailored to fit the defect size and positioned directly over the resection line. The second one was indicated for the prevention or sealing of alveolar air leaks at the visceral pleural surface, applied as a thin and uniform layer over the staple line or areas of detected leakage under sustained inflation of the lung. The standardized volume of Progel applied ranged between 5 and 10 mL per treated site, depending on the extension of the staple line, while Neoveil sheets were used in dimensions of approximately 5 × 5 cm or as required to ensure complete coverage of the target area. Procedures in this cohort were conducted prospectively and in accordance with institutional ethical standards. All patients in both groups provided informed consent for anonymized inclusion in the prospective Epithor database.

### Propensity match score

To reduce selection bias and enhance comparability between groups, a 1:1 propensity score matching was performed using the nearest-neighbor algorithm without replacement, with a caliper width of 0.2 SDsof the logit of the propensity score. Propensity scores were estimated using SPSS (IBM SPSS Statistics, Version 29), based on the following preoperative covariates: type of resection (lobectomy vs segmentectomy), surgical approach (VATS vs RATS), and pulmonary function parameters—forced expiratory volume in one second (FEV_1_), diffusing capacity of the lung for carbon monoxide (DLCO) and chronic obstructive pulmonary disease (COPD). Covariate balance before and after matching was assessed using standardized mean differences, with values <0.1 considered indicative of adequate balance.

### Primary and secondary outcomes

The primary end-point of the study was the length of hospital stay, defined as the number of postoperative days from the first day after surgery until discharge. Hospital discharge was considered appropriate when patients met the following criteria: haemodynamic and respiratory stability (oxygen saturation > 90% on room air or low-flow oxygen), adequate pain control with oral analgesia, tolerance of oral feeding, and satisfactory mobilization with independent ambulation. Chest tube removal was required before discharge, with minimal serous drainage (<400 mL/24 h) and absence of air leak (0 mL/min on Thopaz system) or pneumothorax. Additional criteria included spontaneous diuresis, restored bowel function, absence of unresolved postoperative complications (such as infection, arrhythmia, or bleeding), and patient education regarding home care and warning signs.

Secondary end-points included: duration of chest tube drainage (in days), need for pleural drain reinsertion, requirement for autologous blood patch, 30-day and 90-day hospital readmission rate, incidence of postoperative complications other than prolonged air leak, defined as air leakage from the pleural drain for more than 5 days after surgery (such as pneumothorax, subcutaneous emphysema, atrial fibrillation, pulmonary embolism, postoperative urinary retention requiring bladder catheterization, pulmonary infection requiring antibiotic therapy, atelectasis of the remaining lung, or haematoma at the surgical access site).

### Statistical analysis

Continuous variables were reported as mean with SD, and categorical variables as counts and percentages. Group comparisons were performed using Pearson’s chi-squared test or Fisher’s exact test, as appropriate based on expected cell counts. A two-tailed *P*-value ≤ .05 was considered statistically significant.

Missing data for variables included in the matching procedure and for outcomes were assessed before analysis; given their minimal extent, a complete-case approach was applied.

Covariate balance before and after matching was evaluated using standardized mean differences (SMDs). A Love plot summarizing all SMDs was generated to visually assess improvement in balance after matching. The distribution and overlap of propensity scores between treatment groups were examined to verify the adequacy of the propensity score model and the feasibility of the matching procedure.

## RESULTS

### Patients

**Figure 1.**
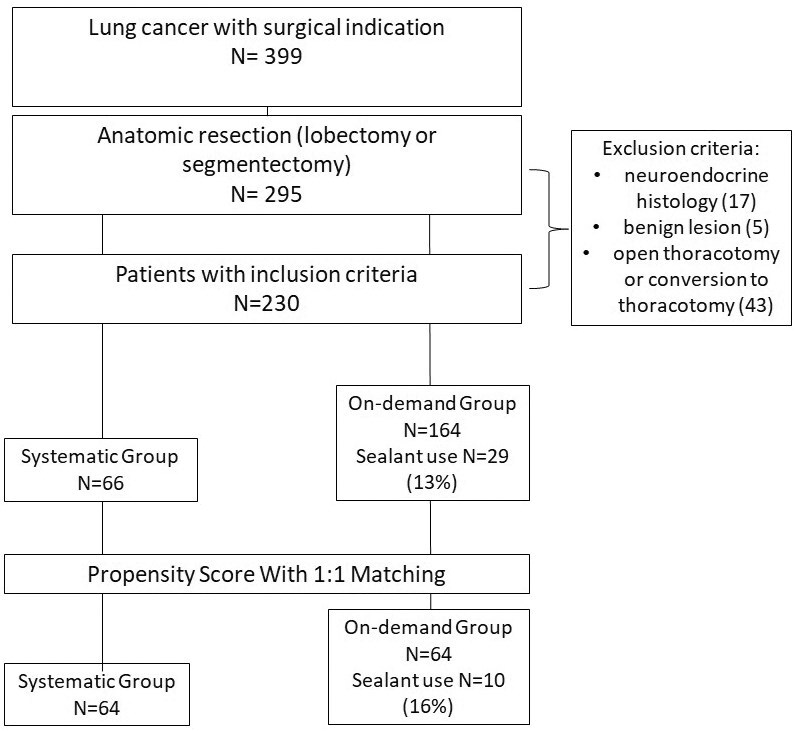
Flow-Chart

A total of 230 consecutive patients were initially identified for inclusion in the study: 164 in the On-Demand Group and 66 in the Systematic Group. After 1:1 propensity score matching, 112 patients were included in the final analysis, with 56 patients in each group (Figure 1). Preoperative characteristics are summarized in Table 1. Before matching, the On-Demand Group had a lower prevalence of COPD, and statistically significant differences were observed in patient age and the type of chest drainage system used. After matching, baseline characteristics between the two groups were generally well balanced (Table 2), with no statistically significant differences remaining. In the On-Demand Group, only 13 patients (23%) received an intraoperative aerostatic patch.

**Table 1.** Baseline Patient Characteristics—Unmatched Patient

Variables		Systematic group (66)	On-demand group (164)	*P*
Age (mean [SD])		69.10 [7,14]	66.06 [11,06]	.036
Sex	Female, *n* (%)	31 (47)	76 (46)	.931
	Male	35 (53)	88 (54)	
Weight kg (mean [SD])		71.2 [14,6]	71.4 [15,0]	.920
Height cm (mean [SD])		168.1 [9,5]	168.1 [8,5]	.962
Smoke	Active, *n* (%)	7 (11)	26 (16)	.261
	Ex-smoker, *n* (%)	47 (71)	98 (60)	
	Never smoker, *n* (%)	12 (18)	40 (24)	
COPD, *n* (%)		25 (38)	26 (16)	.001
Surgery	VATS, *n* (%)	53 (80)	130 (79)	1.000
	RATS, *n* (%)	13 (20)	34 (21)	
Resection	Lobectomy, *n* (%)	46 (70)	108 (66)	.643
	Segmentectomy, *n* (%)	20 (30)	56 (34)	
Air leak test	No, *n* (%)	38 (58)	89 (54)	.648
	Yes, *n* (%)	28 (42)	75 (46)	
Drainage System	Thoraseal/Thopaz, *n* (%)	5 (8)	2 (1)	.031
	Thoraseal, *n* (%)	53 (80)	30 (18)	
	Thopaz, *n* (%)	8 (12)	16 (10)	
	Atmos, *n* (%)	0	2 (1)	
FEV1 (mean [SD])		91.47 [21,68]	90.34 [19,85]	.705
DLCO (mean [SD])		73.41 [18,38]	77.57 [19,26]	.141
Time of surgery (min) (mean [SD])		144.27 [40,69]	146.14 [47,70]	.652

Abbreviations: COPD, chronic obstructive pulmonary disease; DLCO, diffusing capacity for carbon monoxide; FEV1, forced expiratory volume in the 1st second; SD, standard deviation.

**Table 2.** Baseline Patient Characteristics—Matched Patient

Variables		Systematic group (56)	On-demand group (56)	*P*
Age (mean [SD])		69 (7)	67 (9)	.392
Sex	Female, *n* (%)	26 (43)	27 (48)	.705
	Male, *n* (%)	32 (57)	29 (52)	
Weight kg (mean [SD])		71,0 [15,2]	68,2 [13,9]	.190
Height cm (mean [SD])		168,3 [9,3]	166,5 [8,3]	.354
Smoke	Active, *n* (%)	5 (9)	3 (5)	.230
	Ex-smoker, *n* (%)	43 (76)	38 (58)	
	Never smoker, *n* (%)	8 (14)	15 (27)	
COPD, *n* (%)		16 (29)	15 (27)	.270
Surgery	VATS, *n* (%)	43 (77)	46 (82)	.483
	RATS, *n* (%)	13 (23)	10 (18)	
Resection	Lobectomy, *n* (%)	41 (73)	41 (73)	.999
	Segmentectomy, *n* (%)	15 (27)	15 (27)	
Air leak test	No, *n* (%)	34 (61)	31 (55)	.556
	Yes, *n* (%)	22 (39)	25 (45)	
Drainage system	Thoraseal/Thopaz, *n* (%)	4 (7)	0 (0)	.014
	Thoraseal, *n* (%)	45 (80)	8 (53)	
	Thopaz, *n* (%)	7 (13)	7 (47)	
	Missing data	0	41	
FEV1 (mean [SD])		93 [21]	94 [18]	.974
DLCO (mean [SD])		74 [19]	75 [22]	.764
Time of Surgery (min) (mean [SD])		147 [42]	151 [46]	.954

Abbreviations: COPD, chronic obstructive pulmonary disease; DLCO, diffusing capacity for carbon monoxide; FEV1, forced expiratory volume in the 1st second.

SD, standard deviation.

After matching, all covariates showed substantially reduced standardized mean differences, as illustrated in the Love plot (Supplementary Data). The SMD table confirms that all variables reached acceptable balance thresholds. The distributions of propensity scores showed good overlap between groups, supporting the robustness of the matching process. A caliper of 0.12, corresponding to 0.2 SDs of the logit of the propensity score, was applied for nearest-neighbor matching.

### Primary outcome

Primary and secondary outcomes data following propensity score matching are presented in Table 3. The mean length of hospital stay was 7.9 ± 10.8 days (median 5.0 days [IQR, 4.0-8.0]) in the Systematic Group versus 6.2 ± 4.1 days (median 5.5 days [IQR, 3.0-8.0]) in the On-Demand Group. Although there was a trend towards shorter hospitalization in the Systematic Group, the difference did not reach statistical significance (*P* = 0.371).

**Table 3.** Primary and Secondary Outcomes—Matched Patient

Variables		Systematic group	On-demand group	*P*
**Primary outcome**				
Length of hospital stay (mean [SD])		7.9 [10.8]	6.2 [4.1]	0.371
Length of hospital stay (median (IQR 25-75)		5.0 (4.0-8.0)	5.5 (3.0-8.0)	
**Secondaries outcomes**				
Redrainage, *n* (%)		4 (7.1%)	5 (8.9%)	0.999
Complications, *n* (%)		19 (34)	20 (36)	0.491
Prolonged drainage (>5-7 days), *n* (%)		8 (14)	7 (13)	0.781
Blood patch, *n* (%)		3 (5)	2 (4)	0.999
Days of drainage (mean [SD])		4,3 [7,4]	4,7 [3,9]	0.327
Readmission 30-days, *n* (%)		5 (9)	0	0.019
Readmission 90-days, *n* (%)		6 (11)	1 (2)	0.113
Discharge	Home, *n* (%)	51 (91)	53 (95)	0.463
	Another facility, *n* (%)	5 (9)	3 (5)	
	In-hospital mortality, *n* (%)	0 (0)	0 (0)	

Abbreviation: SD, standard deviation.

### Secondary outcomes

Secondary outcomes are detailed in Table 3. Most outcome measures were comparable between the 2 groups and did not reach statistical significance. One notable exception was the hospital 30-days and 90-day readmission rate: respectively 9% and 11% in the Systematic Group versus 2% in the On-Demand Group. However, only 2 of the 6 readmissions in the Systematic Group were directly attributable to postoperative air leak. The remaining 4 were distributed as follows: 1 due to pain decompensation, 2 for cardiovascular events in the first 30 days after discharge and the last one, at 90-days to discharge, for dyspnoea caused by pleural effusion subsequently treated with thoracentesis; these readmissions were unrelated to the surgical procedure and were therefore not considered procedure-specific complications. In the analyzed patient cohort, no in-hospital death was recorded.

## DISCUSSION

Postoperative air leak remains one of the most common and clinically significant complications following pulmonary resection. It is a major contributor to prolonged hospitalization, increased risk of postoperative infections, and reduced quality of life. As minimally invasive approaches have become standard practice in thoracic surgery, the prevention and management of air leaks have assumed critical importance in optimizing clinical outcomes and healthcare resource utilization.^1–4^

This study evaluated 2 intraoperative strategies for air leak prevention following anatomical lung resection for non–small cell lung cancer. The first strategy—a selective, “on-demand” approach—involved the application of aerostatic sealants based on specific intraoperative findings, such as a positive hydro-pneumatic test, fragile lung parenchyma, or dense pleural adhesions, relying on the surgeon’s clinical judgement to assess risk. The second strategy—a systematic approach—entailed the routine application of sealants to anatomical sites considered at high risk for leakage (e.g., parenchymal suture lines or bronchial stumps), regardless of intraoperative evidence of vulnerability.

Our findings indicate that systematic, prophylactic application of aerostatic sealants does not confer significant benefit in short-term clinical outcomes compared to selective, intraoperative assessment-guided use. Length of hospital stay and most secondary end-points were similar between groups after propensity score matching, supporting selective application as both clinically sound and economically sustainable. This challenges the prevailing assumption that routine sealant use reliably prevents postoperative air leaks and associated complications.^5–7^

Notably, the 90-day hospital readmission rate was higher in the systematic sealant group. However, only 2 of 6 readmissions were directly related to postoperative air leaks (pneumothorax detected during outpatient follow-up), with the remainder unrelated to surgery. This finding raises concerns about the long-term effectiveness of routine sealant use. Possible explanations include premature discharge due to a false sense of security or sealants masking subclinical leaks that manifest after discharge.^8^ Furthermore, economic considerations are increasingly critical. Systematic sealant application increases surgical material costs without proportional improvement in patient outcomes. In the context of constrained healthcare resources, interventions must be critically evaluated for cost-effectiveness alongside clinical efficacy.^9^^,^^10^ At last, aerostatic materials may complicate re-operations by obscuring anatomical planes and hindering identification of vital structures, although this was not directly investigated in our study.^11^

A key strength of this investigation is its innovative methodological approach. Unlike prior studies that primarily compare different hemostatic agents based on material properties or isolated clinical outcomes,^13–17^ this study uniquely contrasts 2 distinct application strategies: systematic versus selective, intraoperative assessment-guided use. This shift from product-focused to procedural optimization provides valuable insights for clinical decision-making and tailoring hemostatic agent use according to surgical context.

However, limitations must be acknowledged. The retrospective design carries inherent risks of selection bias, despite use of propensity score matching to improve group comparability. The chronological shift from on-demand to systematic application introduces potential temporal bias, even if balanced by the very short period of inclusion, as evolving perioperative practices or surgeon experience may influence outcomes. Although the 2 cohorts were collected at different time periods, the temporal gap between them is very short (less than 1 week), making meaningful changes in clinical practice and surgical technique highly unlikely. Given this minimal delay, the risk of temporal or learning-curve bias is negligible, and additional time-adjusted analyses were therefore not deemed necessary.

Different types of sealants were employed in each group—polyglycolic acid patches in the on-demand group and polyethylene glycol-based sealants in the systematic group—which could represent a potential source of bias, as these materials differ in both their mechanism of action and their efficacy in preventing postoperative air leaks. Polyglycolic acid patches primarily provide mechanical reinforcement of the staple line, while polyethylene glycol-based sealants act as chemical barriers forming an adhesive hydrogel layer. The selection of these products was not based on surgeon preference or case characteristics, but rather on the hospital’s pharmaceutical policy, which determined product availability during the study period. Finally, additional external validation is necessary before drawing definitive conclusions.

The relatively small matched sample size (112 patients) limits statistical power to detect subtle but clinically relevant differences. Lastly, the absence of a formal economic analysis leaves the cost-effectiveness question unaddressed.

Another potential limitation of the study pertains to the patient’s discharge disposition. Specifically, discharge to a rehabilitation facility may have influenced the duration of hospitalization. In particular, as reported in Table 3, some patients were discharged to rehabilitation facilities, and this modality may have influenced the discharge timing.

## CONCLUSION

These results suggest that selective, intraoperative assessment-guided application of aerostatic sealants is as effective as routine, systematic use in preventing postoperative air leaks after minimally invasive lung resection for NSCLC. Considering the absence of a clear clinical benefit associated with systematic application, a tailored, evidence-based approach is recommended. Prospective randomized trials with larger patient populations and formal cost-effectiveness evaluations are needed to confirm these findings and guide standardized clinical practice.

**Figure 2.**
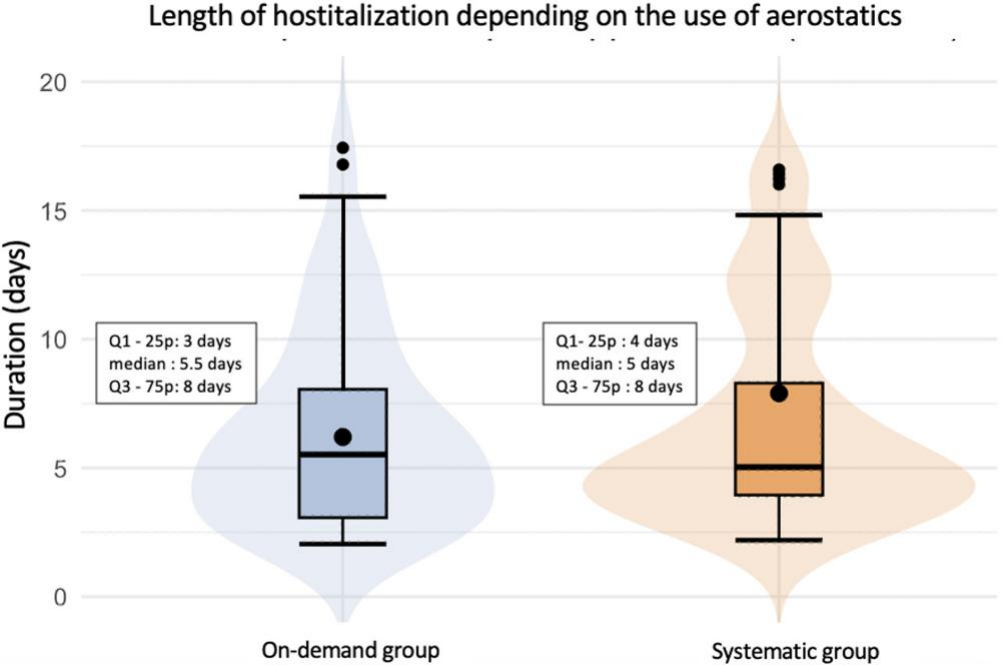
Graphical Representation of the Primary Outcome. The Point in the Box is the Mean, the Bar in the Box is the Median. The Points Above and Below Boxes Are the Extremes of the Population

## Supplementary Material

ivag094_Supplementary_Data

## Data Availability

All data generated or analysed during this study are included in this article and in its online supplementary material. The data underlying this article will be shared on reasonable request to the corresponding author.
