# Supplementary material for: Systematic or On-Demand Sealant Use in Minimally Invasive Lung Surgery: A Matched Comparison
Source: Interdiscip Cardiovasc Thorac Surg. 2026 Mar 28;41(5):ivag094. doi: 10.1093/icvts/ivag094 (PMC13176766; doi:10.1093/icvts/ivag094)
Supplement: ivag094_Supplementary_Data [file ivag094_supplementary_data.docx]

**Supplementary matherials**

Figure S1: Love plot displaying the standardized mean differences (SMDs) before and after matching

*
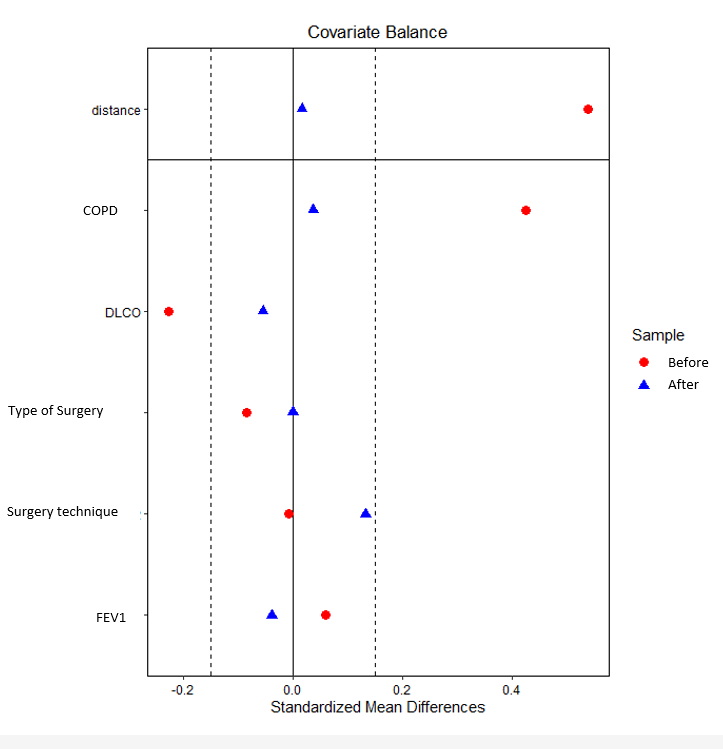
*

Figure S2: Propensity score distribution


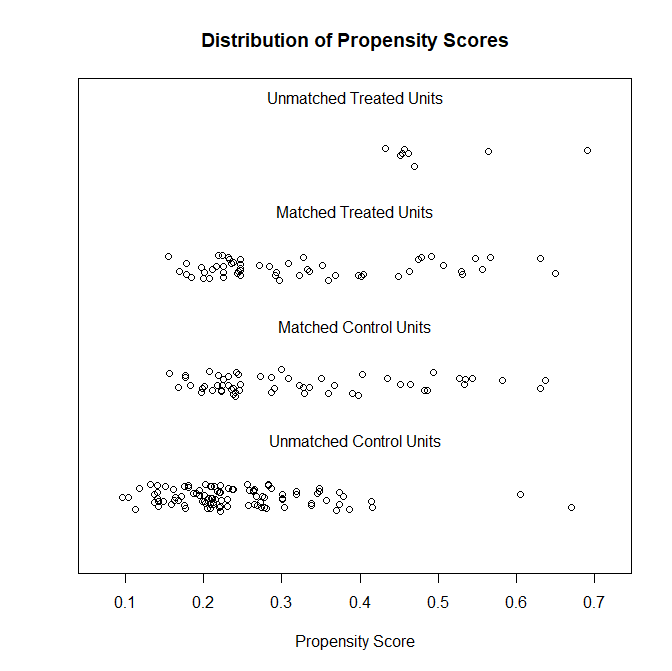


Figure S3: Boxplot showing the comparison between the two groups in the population before and after the propensity score

*
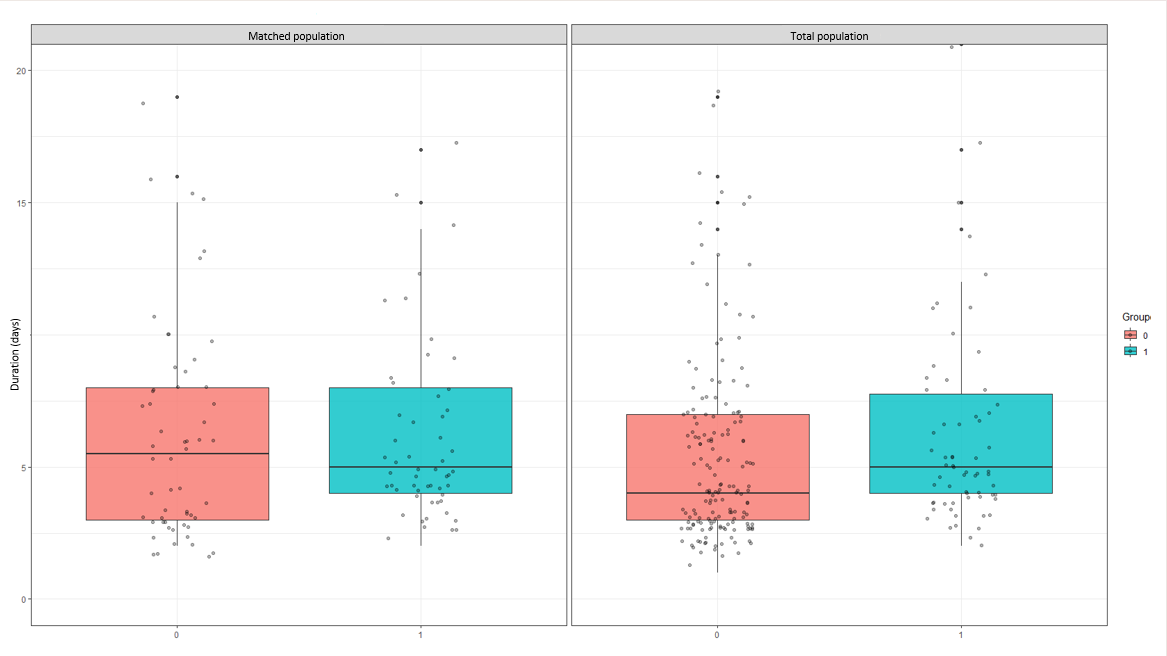
*

Group 0: On-Demand Group

Group 1: Systematic Group

Table S1: Number of missing data and percentage of missing data for the matching variables and outcomes

| **Variables** | **n_NA** | **pct_NA** |
| --- | --- | --- |
| Aerostatics | 0 | 0.00 |
| Type of Surgery | 0 | 0.00 |
| Surgical technique | 0 | 0.00 |
| FEV1 | 2 | 0.87 |
| DLCO | 11 | 4.78 |
| COPD | 0 | 0.00 |
| Length of hospital stay | 1 | 0,43 |
| Days of drainage | 1 | 0,43 |
| Length of surgery | 0 | 0,00 |

n_NA: number of missing data

pct_NA: percentage of missing data

FEV1: Forced Expiratory Volume in the 1st second

DLCO: Diffusing capacity for carbon monoxide

COPD: chronic obstructive pulmonary disease

Table S2: Table of SMD values ​​before and after matching (caliper 0.2*logit) - Standardized mean differences before and after matching

| **Variables** | **SMD before matching** | **SMD after matching** |
| --- | --- | --- |
| distance | 0.539 | 0.017 |
| Type of Surgery | 0.085 | 0.000 |
| Surgical technique | 0.008 | -0.133 |
| FEV1 | 0.060 | -0.039 |
| DLCO | -0.227 | -0.054 |
| COPD | -0.426 | -0.037 |

SMD: standardized mean differences

FEV1: Forced Expiratory Volume in the 1st second

DLCO: Diffusing capacity for carbon monoxide

COPD: chronic obstructive pulmonary disease

- Figure S1: Love plot displaying the standardized mean differences (SMDs) before and after matching
- Figure S2: Propensity score distribution
- Figure S3: Boxplot
- Table S1: Number of missing data and percentage of missing data for the matching variables and outcomes
- Table S2: Table of SMD values ​​before and after matching (caliper 0.2*logit) - Standardized mean differences before and after matching
